# An Asymptomatic Patient with an Additional Cardiac Chamber Giant Left Atrial Appendage

**DOI:** 10.1155/2020/6519089

**Published:** 2020-02-07

**Authors:** Alexandros P. Evangeliou, Evangelia Sotiroglou, Nikolaos Charitakis, Georgios Loufopoulos, Christos Varassas, Spiridon Papadopoulos, Stergios Tzikas, Vassilios Vassilikos

**Affiliations:** ^1^3rd Department of Cardiology, Ippokratio Hospital, Aristotle University of Thessaloniki, Thessaloniki, Greece; ^2^Department of Radiology, Ippokratio Hospital, Thessaloniki, Greece

## Abstract

We present the case of an asymptomatic 54-year-old male, referred to our department for a follow-up cardiological consultation. Echocardiography assessment showed an unknown cavity adjacent to the lateral wall of the left ventricle. A large left atrial appendage was revealed in further investigations, and the treatment option was proved to be an impasse.

## 1. Introduction

Giant LAA or left atrial appendage aneurysm (LAAA) is an uncommon finding, and it either has a congenital origin or is acquired due to valvular dysfunction such as mitral regurgitation. Most of the cases are asymptomatic, but systemic thromboembolic events and supraventricular tachycardias (SVTs) may also be complications of untreated patients.

## 2. History of Presentation

A 54-year-old male presented at our department for follow-up consultation a year after percutaneous coronary intervention for an acute coronary syndrome involving the right coronary artery. The patient was asymptomatic, denying angina, palpitations, or other cardiac symptoms. The clinical examination, as well as the laboratory blood measurements, showed no abnormality.

## 3. Past Medical History

The past medical history of the patient comprises arterial hypertension, an acute myocardial infarction due to the right coronary artery obstruction, managed with interventional implantation of a drug-eluting stent in the culprit lesion, and a hospitalization due to a pleural empyema following a pulmonary infection a decade ago.

## 4. Investigations

Twelve-lead electrocardiogram confirmed sinus rhythm, atrioventricular conduction was normal, QRS complex width was narrow, the repolarization was typical, and the absence of supraventricular arrhythmia or other structural heart diseases is appreciated.

**Figure 1:**
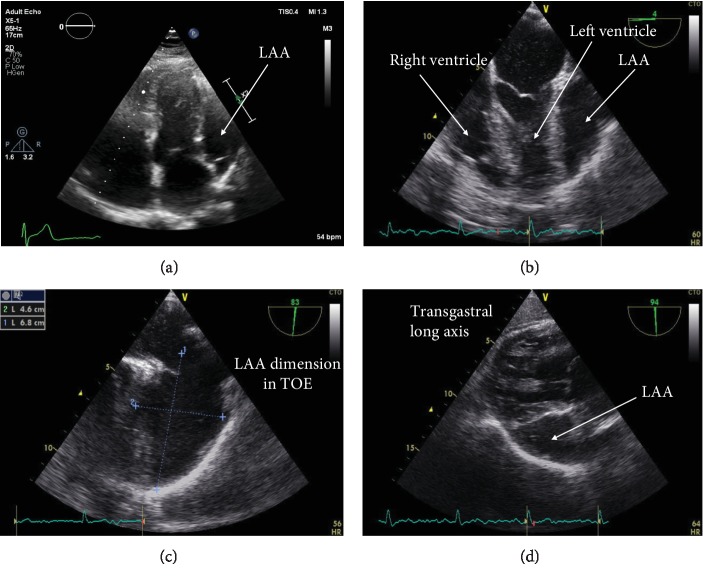
Echocardiographic views of the LAA.

The transthoracic echocardiography (TTE) assessment unveiled normal functional features and no valvular dysfunction, and a cavity without contractility adjacent to the basal lateral wall of the left ventricle was notable (Figure 1(a), video ([Supplementary-material supplementary-material-1])). Due to the inability to accurately characterize this echocardiographically depicted abnormality, further medical investigations were performed. A transesophageal echocardiography (TEE) assessment (Figures 1(b)–1(d)) showed communication of the cavity with the left atrium with slow blood flow within the LAA without thrombi; thus, the diagnosis of the giant LAA was established. The LAA was undiagnosed in the TTE assessment which was performed one year ago, since it is an entity which cannot be easily revealed and further investigation is required.

**Figure 2:**
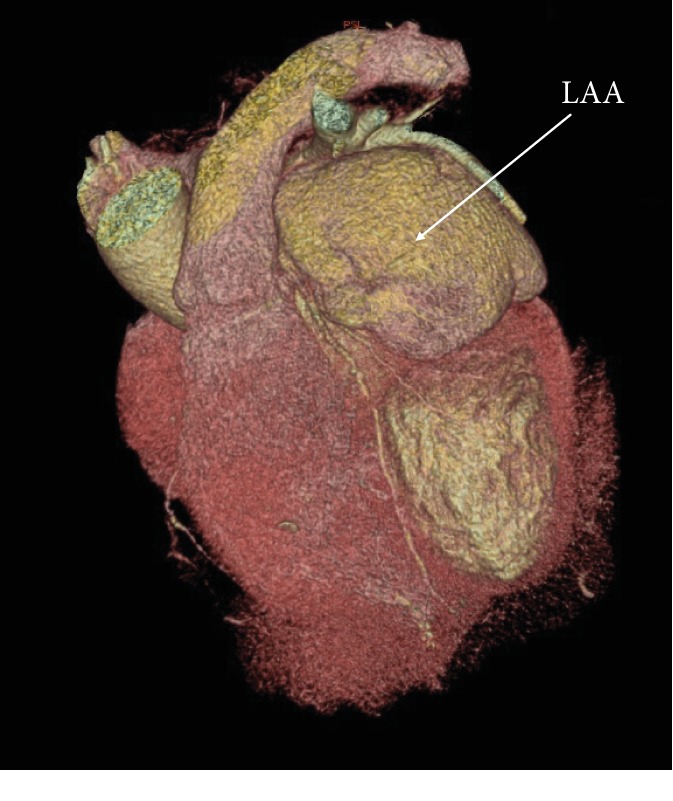
Cardiac CT depicting the LAA. Angiographic “shadow” of the LAA.

Cardiac computed tomography (CT) was performed to evaluate the size, the relation of LAA with around organs, and the communication with the left atrium. CT revealed an abnormal left border of cardiac silhouette and enlargement of LAA (Figure 2). The enhancement with the contrast agent confirmed the presence of a LAA (4.6×6.8 cm in size).

In order to assess the possible systemic thromboembolic risk, considering the size of the LAA, a brain magnetic resonance imaging (MRI) was performed. T2 and fluid-attenuated inversion recovery (FLAIR) brain MRI revealed white matter hyperintensity (WMH) lesions in both cerebral hemispheres, without enrichment of lesions after the contrast agent administration as a sign of prior cerebral embolisms.

## 5. Differential Diagnosis

The differential diagnosis comprises pleural effusion, pericardial effusion, giant LAA, and LAAA with an acquired origin.

## 6. Management

Due to the lack of evidence-based guidelines for the management of such cases, our prime consideration was to assess the thromboembolic risk of this congenital abnormality. The idea to interventionally seal the LAA, in order to minimize the thromboembolic risk, is futile due to the large LAA neck (7.5 cm^2^, maximal diameter > 40 mm). The available occluders with a maximum diameter of 33 mm are inappropriate in this case. Moreover, the absence of symptoms or echo contrast inside the LAA also excludes surgical treatment. However, after the MRI findings, oral anticoagulation was proposed as a possible management, and the patient accepted this option of treatment.

## 7. Discussion

Giant LAA or LAAA is an uncommon finding, and only a few cases are reported on the published literature. There is no consensus about these two medical terms, and most of the time, an extensive misunderstanding exists, concerning the description of a huge LAA in echocardiography assessment. It either has a congenital origin or is acquired due to valvular dysfunction such as mitral regurgitation [1]. Congenital LAA is mostly asymptomatic, but systemic thromboembolic events and SVTs due to atrial enlargement may also be complications of untreated patients [2]. LAA is the most likely site of thrombus formation in patients with atrial fibrillation (AF), indicating a clinically significant early diagnosis.

The treatment armamentarium for patients with giant LAA includes surgical aneurysmectomy or ligation [3] and oral anticoagulant for prophylaxis of systemic thromboembolic events as well as antiarrhythmic therapy for atrial tachyarrhythmias when indicated [4, 5]. Recommendation of surgical occlusion in symptomatic patients is based only on nonrandomized or observational cohort studies [6], as it can prevent thromboembolic complications and atrial arrhythmias associated with the LA enlargement.

Transcatheter device occlusion [7] has also been reported for the occlusion of narrow neck LAAs, with a high rate of success, in patients with anticoagulant contraindication [8]. All the published case reports are referred to the occlusion of atrial appendage with an acceptable width of the neck while the available sizes of these occluders are not larger than 33 mm. So, there is no report of device occlusion in really giant LAA treatment.

There is a gap of evidence in the management of asymptomatic and uncomplicated patients with normal sinus rhythm and huge LAA. Some authors favor surgery even in asymptomatic patients to prevent possible morbid and fatal complications associated with AF and systemic embolization [9]. In addition, there is no evidence that treatment of asymptomatic patients with oral anticoagulant agents can lead to stroke prevention, overwhelming the adverse events of such treatment. Interventional LAA occlusion is not an option in the majority of cases, due to device LAA orifice mismatch.

As far as our patient is concerned, despite being asymptomatic, his brain MRI demonstrated WMH lesions in both cerebral hemispheres, showing an increased risk of future systemic thromboembolic events [10]. However, the absence of arrhythmia, symptomatic stroke, or transient ischemic attack could not empower the choice of surgical excision, thus leaving anticoagulation as the only reasonable alternative.

## 8. Follow-Up

The patient remains asymptomatic and uncomplicated during the 3-month follow-up period. There are no signs of cardiac arrhythmias or systemic embolization, he remains in sinus rhythm, and no additional hospitalizations have occurred.

## 9. Conclusion

Giant LAAs can be initially depicted as an additional cardiac chamber. Further imaging can reveal the complete anatomy of this mostly congenital anomaly. In asymptomatic patients with giant LAAs, surgical occlusion is not recommended, and no evidence-based guidelines exist so far. After assessing the individual thromboembolic and bleeding risk of the patient, an appropriate treatment option might be oral anticoagulation therapy.

## 10. Learning Objectives

The following are the learning objectives:

1. To be able to recognize the presence of a LAA with TTE or TEE examination in the asymptomatic patient because most of the times they are misdiagnosed as a pericardial fluid
2. To be able to define the right treatment option for this patient if the transcatheter or surgical occlusion is indicated
3. To be able to determine the appropriate treatment option, depending on the existence and the progression of clinical symptoms of the patient during the follow-up period

## Abbreviations

LAA: Left atrial appendage
LAAA: Left atrial appendage aneurysm
SVTs: Supraventricular tachycardias
TTE: Transthoracic echocardiography
TEE: Transesophageal echocardiography
CT: Computed tomography
MRI: Magnetic resonance imaging
FLAIR: Fluid-attenuated inversion recovery
WMH: White matter hyperintensity
AF: Atrial fibrillation.

## Supplementary Materials

Supplementary Materials The transthoracic echocardiography (TTE) assessment unveiled a cavity without contractility adjacent to the basal lateral wall of the left ventricle.
